# Clinical implications of lipid peroxidation in acne vulgaris: old wine in new bottles

**DOI:** 10.1186/1476-511X-9-141

**Published:** 2010-12-09

**Authors:** Whitney P Bowe, Alan C Logan

**Affiliations:** 1 Department of Dermatology, State University of New York Downstate Medical Center, Brooklyn, New York, 11203, USA; 2 Integrative Care Centre of Toronto, 3600 Ellesmere Road, Unit 4, Toronto, ON M1C 4Y8, Canada

## Abstract

Acne vulgaris is a common dermatological disorder, one that is frequently associated with depression, anxiety and other psychological sequelae. In recent years there has been an increasing focus on the extent to which oxidative stress is involved in the pathophysiology of acne. Emerging studies have shown that patients with acne are under increased cutaneous and systemic oxidative stress. Indeed, there are indications that lipid peroxidation itself is a match that lights an inflammatory cascade in acne. The notion that lipid peroxidation is a 'starter gun' in acne is not a new one; here we review the nearly 50-year-old lipid peroxidation theory and provide a historical perspective to the contemporary investigations and clinical implications.

In addition, we present a novel hypothesis in which lipid peroxidation may be priming an increased susceptibility to co-morbid depression and anxiety in those with acne. The emerging research on the systemic burden of oxidative stress in acne sheds further light on the brain-skin axis. The recent findings also suggest potential avenues of approach for the treatment of acne via specific nutrients, dietary modifications, oral and topical interventions.

## Introduction

Acne vulgaris is a common disease in developed nations, one that has increased in frequency in the last half century, particularly among adult women [1]. While the experience of acne may not be life threatening per se, it does carry with it significant psychological disability. Indeed the psychological sequela of acne includes higher rates of clinical depression - up to three times higher than the general population [2]. Higher levels of anxiety, anger, suicidal thoughts and even suicide itself have been noted [3-7]. Evaluations have determined that patients with acne have a more significant impairment of mental health than many other chronic medical conditions, including epilepsy and diabetes [8].

Despite technological advances and an increased degree of sophistication within experimental dermatology, the precise mechanisms of the acne process remain elusive. In general terms, acne is characterized by sebum overproduction, follicular hyperkeratinization, and an increased release of inflammatory-mediating chemicals. Androgens, microbes and other pathogenetic influences are also at work in the development of acne [9,10].

In the past, it was thought that follicular plugging (comedones) preceded *Propionibacterium acnes *(*P. acnes*) colonization, which subsequently resulted in inflammation (papules and pustules). This sequence of events has been called into question in recent years. It has been discovered that subclinical inflammatory events are occurring in acne-prone skin even prior to hyperproliferative and abnormal differentiation events [11,12]. The reason for elevated pro-inflammatory factors, such as interleukin-1 (IL-1), around the clinically normal pilosebaceous follicles of acne patients remains unknown. At this point it simply highlights that the release of inflammatory chemicals is indeed one of the earliest events to occur in the acne process. Furthermore, oxidative stress within the pilosebaceous unit alters the environment from one that is unsuitable to harbor anaerobic bacteria to one that is perfectly suited for the colonization of such species [13]. *P. acnes*, once thought to be the initiating factor of inflammatory acne, might never make the pilosebaceous unit its home were it not for this initial inflammatory insult to the sebum. Oxidation of sebum alters oxygen tension in the follicle, resulting in the micro-aerophilic environment required for P. acnes to survive. Apparently, inflammation and oxidative stress might set the stage for all subsequent pathogenic factors leading to acne.

## Lipid Peroxidation and Acne - Early Research

One plausible mechanism driving the early release of inflammatory mediators is that described by the near half-century-old lipid peroxidation theory of acne. In 1965, University of Chicago investigator Allan L. Lorincz postulated that oxidative breakdown of squalene and other skin lipids may not merely be a consequence of the acne process. He suggested that lipid peroxides might be directly 'acnegenic to the skin'. Based on his theory, it was hypothesized that antioxidants would be of value in limiting and preventing the condition via reduction in the formation of peroxides and other oxidation products. In a small controlled pilot study (n = 15) he reported clinical success with topical alpha-tocopherol (0.05%) in acne after one month of evaluation by an independent examiner [14]. While there had been previous reports of clinical success with vitamins A and C [15-19], Lorincz was the first to specifically consider antioxidants to be of value in acne. The previous benefits of what would turn out to be called antioxidant vitamins and flavonoids (including an 800 mg oral vitamin C and hesperidin complex) were largely attributed to support of normal keratinization [17,18].

Further support to the lipid peroxidation hypothesis would come from University of California, Davis, scientist Alloys L. Tappel, who reported in 1975 that lipid peroxidation is evident in acne, and that localized free radical damage and peroxides might be involved in initiating the damaging inflammatory reactions [20]. Following this, other investigators reported that components of sebum, particularly squalene, show enhanced comedogenicity when oxidized [21]. Indeed, squalene was reported to be highly sensitive to oxidation and researchers reported that both squalene and its oxidized metabolites are found at much higher levels in acne vs. healthy controls [13,22-24]. University of California, Los Angeles, dermatologist Samuel Ayres Jr. and colleague Richard Mihan provided observational case reports indicating benefit of the synergistic value of oral vitamins A and E as a means to attenuate lipid peroxidation in acne. They urged investigators to pursue the lipid peroxidation hypothesis, suggesting that inflammation in acne is a secondary event to lipid peroxidation. In 1978 Ayres and Mihan commented 'that the administration of an antioxidant might be a logical and effective means of controlling the inflammation by preventing oxidation of lipids' and 'the subsequent release of irritating free radicals into the tissues' [25]. This was followed by preliminary reports of low blood levels of glutathione peroxidase (GSH-Px), and its required mineral co-factor selenium, among patients with acne [26-28]. It was also noted, a quarter century ago, that the therapeutic value of tetracycline antibiotics in acne might be due to their ability to act as antioxidants [29]. These early findings and the urging of Ayres and Mihan did not, however, advance the lipid peroxidation theory into mainstream dermatological discussions.

## Contemporary Investigations - Systemic Oxidative Stress

In recent years there has been renewed interest in the influence of oxidative stress and the operations of the antioxidant defense system in acne. Many of these investigations have examined the extent to which a potential oxidative stress burden in the skin might be reflected in the blood of acne patients. In a study involving 52 patients with papulopustular acne, researchers reported that blood antioxidant enzyme activities, including superoxide dismutase (SOD) and GSH-Px, were significantly lower vs. controls [30]. Presumably, the decreased activity is a result of the consistent burden of oxidative stress. As we will discuss, the consumption of antioxidants and the demand for antioxidant enzymatic activity may not be a mere consequence of the downstream clinical manifestations of acne. The lower blood GSH-Px activity replicated the initial work of Michaelsson and Edqvist 17 years earlier [27]. In addition, there was evidence of an increased oxidative stress burden as reflected in higher thiobarbituric acid reactive substance (TBARS) levels in the acne patients. Separate investigations have also found elevated malondialdehyde (MDA) [31], a reactive species well known to be a marker of increased oxidative stress. Emerging studies indicate that low blood SOD, GSH-Px and elevated MDA are characteristic of acne vs. healthy controls [32-35]. These differences are not minute; indeed the GSH-Px activity in adults with acne is reported to be 42 percent lower than healthy controls [34]. Some investigators have not found a blanket decrease in SOD and increase in MDA among all acne patients. One investigative group noted that patients with the more severe forms of acne have the lowest SOD and highest MDA levels vs. other milder cases and controls [36].

Hydrogen peroxide is a reactive oxygen species (ROS) produced by neutrophils, and as with other ROS, it is well capable of promoting inflammation, causing tissue damage and further lipid destruction. Hydrogen peroxide production was measured from whole blood samples taken from adults with inflammatory acne vs. healthy controls. Patients with inflammatory acne had significantly higher production of hydrogen peroxide, 43% more than healthy controls. After treatment with minocycline there was a 25% reduction in whole blood, microbial and chemically stimulated, neutrophil hydrogen peroxide production. Since the researchers used both *Staphylococcus aureus *and phorbol myristate acetate to stimulate the blood neutrophils (there was no difference in neutrophil response to microbes vs. chemical stimulation in acne), it appears that the ROS generating system is primed for accelerated production in acne [37]. Also, since minocycline and other tetracycline antibiotics are now known to have clear antioxidant properties, particularly in reducing lipid peroxidation in potency similar to vitamin E [38], the results lend further support to the suggestion - 17 years earlier - that this class of antibiotics may be helping via non-antimicrobial pathways in reduction of ROS. Indeed, a recent investigation showed that doxycycline, at sub-antimicrobial doses, reduced papules and pustules by over 80% after 3 months in those with moderate facial acne [39].

Some of the emerging studies have also measured plasma and serum levels of antioxidant nutrients in acne patients. Among 100 newly diagnosed and untreated acne patients, plasma levels of vitamins A and E were significantly lower than controls. Indeed, the severity of acne showed a negative correlation with blood antioxidants, such that the vitamin A levels in those with the most severe acne were 52% lower and the vitamin E levels were 31% lower vs. healthy age-matched controls [40]. Low serum levels of vitamins A and E, as well as beta-carotene and vitamin C, have also been reported in a group of 45 female acne patients vs. healthy controls (respectively vitamin A 33%, vitamin C 40%, vitamin E 46%, beta-carotene 64% lower on average) [41]. Low serum levels of vitamin A in untreated acne patients had been previously documented in 1978 [42]. The contemporary investigation also added in an additional blood enzyme as an end-point - platelet monoamine oxidase (MAO) - the activity of which has been implicated in a variety of mental health disorders, including anxiety and depression. The investigators found low MAO activity in acne, a finding which, while not universal, is consistent with previous investigations in affective disorders [43]. As we will discuss shortly, this finding has enormous implications in the lipid peroxidation theory of acne and its overlap with the emerging research showing oxidative stress is not a mere consequence of mental health disorders.

## Emerging Research within Experimental Dermatology

Although the recent studies on blood markers of oxidative stress in acne are of importance, they are limited in that they provide no insight into oxidative stress as a causative agent or mere consequence of the disease. The findings of increased systemic oxidative stress and diminished blood levels of antioxidants in acne take on greater meaning when placed in context of the new findings within experimental dermatology. A series of sophisticated bench studies have pointed to lipid peroxidation as the match that lights the inflammatory process in acne.

In support of previous research, it has become increasingly clear that squalene production is highly upregulated in acne. Overall acne patients have 59% more sebum than healthy controls, yet it is squalene that emerges as the specific lipid that is being produced in abundance - 2.2-fold higher vs. controls [44]. As expected, an increase in squalene sets the stage for significantly higher levels of squalene peroxides and diminished vitamin E in the sebum of acne patients [45]. Squalene peroxides also diminish the important skin antioxidant glutathione, while pre-treatment with glutathione depleting agents (DL-buthionine sulfoximine and diethyl maleate) makes the comedogenic potential of squalene peroxides even worse [46]. In smokers with acne the squalene peroxidation and vitamin E reduction is even more pronounced [47]. Not only are the squalene peroxides confirmed to be highly comeodogenic [48], they have recently been reported to set an inflammatory cascade in motion. Specifically, exposure of peroxidated squalene products to human keratinocyte cells stimulates production of inflammatory cytokines and upregulates lipoxygenase (LOX) activity [49]. Considering that LOX activity, and leukotriene B4 (LTB4) in particular, has been implicated in promoting inflammation in acne (even in the absence of *Propionibacterium acnes*), this was an important discovery. LTB4 is a chemoattractant well capable of recruiting ROS-generating neutrophils, and its inhibition has been shown to improve acne in clinical research [50,51].

Researchers have also had a closer look at the mechanisms through which *P. acnes *promotes inflammation in acne. Once again, it appears rooted in the initial production of ROS. When keratinocytes are exposed to *P. acnes *surface proteins there is an immediate generation of ROS, most notably superoxide. The superoxide is handled by SOD which converts it into hydrogen peroxide, and then GSH-Px is called upon to dispose of the hydrogen peroxide by converting it into water [52]. Obviously, this study can provide rationale as to why SOD and GSH-Px might become exhausted due to the burden of oxidative stress, particularly in more severe forms of acne. In addition, the researchers discovered that drugs and nutrients with anti-acne activity - isotretinoin, retinol, zinc sulfate - showed significant inhibition of the *P. acnes*-stimulated superoxide production. The authors conclude, as others have before, that inhibition of inflammation 'using appropriate antioxidant molecules could be considered as potential treatment of acne' [52].

Additional research seems to confirm that lipid peroxidation is the driving force behind the progression of comedogenesis and inflammation in acne. Examination of comedo samples (20-30 comedones from each patient) removed from acne patients shows that lipid peroxidation is evident even in the earliest microcomedo. As the disease progresses to inflamed lesions there is an up to 4-fold increase in lipid peroxide levels [53]. The marked increase in lipid peroxidation once inflammation is ongoing is to be expected. Undoubtedly ROS can provoke the secretion of inflammatory cytokines; however, once initiated, inflammatory chemicals cause a subsequent increase in ROS production [54]. Agents with anti-inflammatory activity are important in the reduction of ROS activity, yet the new findings suggest that antioxidants may provide therapeutic efficacy closer to the root.

## Lipid Peroxidation, Acne and Mental Health

As mentioned previously, the rates of depression and anxiety in acne are much higher than that of healthy controls. It would be simple to write this off as a consequence of a disease that, in most cases, presents itself in such a visible way. Such a view is largely justifiable. There is no question that the development of acne, or any visible skin disease, can induce depressive symptoms and anxiety, particularly social anxiety. Yet, might it also be true that mood-related symptoms pre-date or even set the stage for a higher propensity to acne? Might the physiological pathways involved in promoting susceptibility to acne also promote a greater generalized risk of mental health disorders? Why is it that despite marked clinical success with topical and oral interventions, a number of studies using validated measurements of depression, mood and quality of life, indicates that the mental outlook remains unchanged [55-58]? Indeed, in one of the studies cited [55], mood scores declined despite significant clinical improvement with topical interventions. In a systematic review examining depression and isotretinoin, only 1 out of 4 studies using validated depression instruments showed a statistically significant reduction in depressive symptoms [59]. It seems remarkable that an agent with such obvious clinical benefit would only show a trend toward improving depressive symptoms and mental outlook. Some dermatologists have pondered this paradox over the years, as did William O. Roop who, after detailed review of clinical histories, commented in 1921 that 'I am led to believe that this depression precedes the acne, and is more of a causal factor than it is a resultant of the disease' [60]. Some groups have focused on the influence of life stressors in acne development or exacerbation [61]. It has been postulated that differences in personality features which predate the onset of acne might lead to greater stress reactivity and vulnerability to acne [62-65].

Famed British physician Sir Charles Blackburn theorized in 1951 that unknown endogenous factors may be driving higher rates of psychoneurogenic disturbances in acne [66]. While acknowledging that the disease itself can undoubtedly cause mental distress, his contention was that certain groups of acne patients 'must have a previous liability' to mental health disorders, and that such liability may be acted upon by internal factors that have yet to be determined. The end result, according to his liability theory, was an increased risk of diagnosable mental health disorders in acne. With this background, the new findings in the area of lipid peroxidation and acne cannot be viewed in isolation, particularly since a similar line of evidence continues to mount in the field of mental health, one linking lipid peroxidation with depression and anxiety [67]. It is our hypothesis that oxidative stress may be a key endogenous factor causing a pronounced liability to anxiety and depression among acne patients.

If there is enhanced systemic oxidative stress and lipid peroxidation in acne, and the serum/plasma human studies discussed above can only suggest that this is indeed the case, there would be every likelihood of changes to mitochondrial function, membrane fluidity and changes to enzymes and ion channel functioning within the nervous system. Oxidative stress can also compromise normal production of brain growth factors, levels of which have been inversely associated with risk of depression and anxiety. Even small increases in oxidative stress, and lipid peroxidation in particular, may be a causative factor in depression and anxiety [68-70]. Oxidative stress may not be a mere consequence of mental health disorders, as recent experimental studies show that intentional induction of oxidative stress can significantly increase behavioral changes indicative of anxiety [71]. Induction of oxidative stress also increases blood insulin levels, an additional factor pertaining to acne and depression which we will discuss shortly.

We are struck by the similarity (vs. acne) of greater demands upon SOD enzymes and GSH-Px activity, as well as elevated MDA in the periphery of patients with affective disorders [72,73]. Blood levels of antioxidant nutrients have been shown to be low in clinical depression [74]. We also find it noteworthy that two antioxidant co-factoring minerals - zinc and selenium - are low in both acne and depression [75-77]. It hints further at an increased nutrient demand in conditions related to systemic oxidative stress. Indeed, zinc has been the subject of recent clinical investigations in both acne and depression and it has been shown to improve outcome in treatment-resistant depression [78,79].

Older studies indicate that oral vitamin D has therapeutic value in acne [80-82], and emerging studies are showing that topical vitamin D analogues have comedolytic properties [83]. It is also of note that vitamin D has antioxidant properties, can increase SOD and GSH-Px activity [84,85], and low levels are associated with symptoms of depression and anxiety [86,87]. While there has been much discussion on the influence of excess vitamin A and isotretinoin in mood disturbances, it is also evident that vitamin A deficiencies can result in depressive behaviors [88]. Vitamin C has also been recently reported to have anti-depressant activity in experimental settings and epidemiological investigations [89,90]. Keeping in mind that the human studies discussed above showed that untreated patients with acne have low blood levels of vitamin A and C, a connection to depression cannot be dismissed.

Over the last few years it has become clear that anti-depressant agents, including various serotonin reuptake inhibitors (SSRIs), possess ROS-scavenging properties and prevent declines in SOD activity in conditions of increased lipid peroxidation [91-93]. A recent human study has shown that oral supplementation with a microencapsulated SOD improved cognition, irritability and mental outlook vs. placebo after four weeks [94]. In addition, experimental studies suggest that dietary plant-based antioxidants can influence neurotransmitter availability and protect against stress-induced behavioral changes [95-99]. Finally, antioxidant components of the diet may protect against depression [100,101], and greater adherence to a Mediterranean-style diet rich in antioxidants has been shown to reduce the 5-year risk of depression by approximately 30 percent [102].

In one population study involving 18-19 year-old students in Norway, researchers reported no connection between 'fatty fish' consumption, acne and mental distress [103]. Unfortunately the authors relied upon dietary recall methods without validated nutritional instruments and did not define 'fatty fish' to the participants - almost certainly why 93 percent of the sample claimed to have consumed fatty fish only on rare occasion. In addition, they found a self-report acne prevalence of only 13.6 percent for teenage males and females, much lower than would be expected in Western nations. Ultimately the determination of depression and anxiety as risk factors for acne can only be assessed by prospective studies, such as those that have indicated depressive symptoms and anxiety in normal-weight youth can pre-date subsequent risk for later obesity [104,105]. In the meantime, it is our contention that over-lapping lipid peroxidation may be taxing to the antioxidant defense system in both acne and depression. This may tip the scale to further damage and inflammation at the sebaceous gland, and also, at the same time, negatively influence neuronal communication. Ultimately this may set the stage for higher rates of depression and anxiety in acne. Preliminary reports of clinical success in acne treatment with antidepressant medication as monotherapy [106,107] should encourage detailed exploration of the brain-skin axis. It may be the case that certain acne-prone individuals, or a subset of acne patients, are primed for lipid peroxidation long before depression and acne become clinically apparent.

## Clinical Implications

Based on the above discussions, it would seem reasonable that clinical interventions with oral and topical agents designed to support the antioxidant defense system would be helpful in acne. For now, the bench investigations have not stimulated large-scale clinical trials to determine if antioxidants can limit the acne process or augment the value of other first line acne interventions. Although sparse, a few preliminary studies have provided encouraging results and suggest that an internal-topical combination of antioxidants might be the most suitable clinical approach.

An emerging candidate is the stable vitamin C precursor, sodium ascorbyl phosphate (SAP), an agent which has been shown to reduce sebum oxidation products by up to 40%. A preliminary open-label study involving 60 acne patients used 5% SAP (applied twice daily) and showed superior efficacy and tolerance after 12 weeks vs. 5% benzoyl peroxide (BP) [108]. It was also reported in a second open-label study that 5% SAP was more effective than 1% clindamycin in the overall and inflammatory lesion counts after 12 weeks [109]. This was followed up recently by randomized double-blind intervention studies. The first showed that 5% SAP reduced inflammatory acne lesions 49% after 8 weeks, a result that was slightly enhanced by the co-administration of SAP and 0.2% retinol. The combination reduced inflammatory lesions by 63% over the same period [110]. The most recent study showed that 61% of patients treated with 5% SAP lotion showed improvement in the Investigator's Global Assessment Score (IGAS) and 71% improved as measured by the Subjects' Global Assessment Score (SGAS). These results were in contrast with the group receiving the placebo vehicle lotion where the reports were 38% improvement via IGAS and 52% via the SGAS at the end of the 12-week study [111].

Green tea is a source of plant-based antioxidants, particularly the catechins which are well known to prevent local and systemic declines in SOD and GSH-Px activity, as well as attenuating lipid peroxidation [112,113]. A recent clinical study has reported value of a topical (2%) green tea lotion in mild-to-moderate acne. The open-label study showed that twice-daily application of the green tea lotion reduced total lesion count by 58% after 6 weeks [114]. A similar study using a 2% tea (unspecified type of tea) lotion found that it was more effective than 5% zinc sulfate topical preparation in reduction of acne lesions after 8 weeks [115]. It should be pointed out that there are multiple mechanisms whereby green tea may be therapeutic in acne - anti-inflammatory activity, anti-microbial activity against *P. acnes*, and a potential ability to reduce sebum production via 5-α-reductase inhibition [116,117]. In any case, the antioxidant activity of green tea and its catechins warrant further investigation.

Additional support for the use of topical antioxidants is found in a recent open-label trial of the antioxidant agent fullerene. It has been reported that fullerene, a spherical carbon molecule with a unique cage structure capable of acting as a sponge to free radicals, has antioxidant activity several hundred times higher than vitamin-based antioxidants. In particular, fullerene can protect against lipid peroxidation. A 1% fullerene gel applied twice daily reduced the number of inflammatory lesions by 38% during the 8-week investigation. While small (n = 11) and without a placebo control, this pilot investigation in adults with acne suggests potential value of fullerene as a topical antioxidant therapy [118].

Zinc and nicotinamide are both nutrients which support antioxidant pathways [119,120], and preliminary clinical trials show value in topical application. Zinc is well known to enhance efficacy of topical antibiotics, while a 4% nicotinamide gel has been shown to outperform 1% clindamycin gel when applied twice daily for 12 weeks (82% vs. 68% improvement respectively) [121]. There are a limited number of small studies indicating value of oral zinc in acne, the most recent showing that 30 mg of zinc gluconate reduces total inflammatory lesion count by 57% after two months [122]. In the open-label Nicomide Improvement in Clinical Outcomes Study (NICOS) an oral nutrient combination (750 mg nicotinamide, 25 mg zinc, 1.5 mg copper, 500 mcg folic acid) taken daily for eight weeks appeared to be effective and well tolerated. After four weeks 79% of the subjects demonstrated at least moderate improvement, and the addition of oral antibiotic therapy to one subgroup (51 of the total n of 198) did not provide any additional clinical benefit [123].

An oral multi-nutrient antioxidant agent has been the subject of a recent 12-week preliminary trial involving 48 patients with acne. The antioxidant capsule was taken tid for a daily total of 45 mg zinc, 180 mg vitamin C, 18 mg mixed carotenoids, 45 IU d-alpha-tocopherol acetate and 390 mcg of chromium. Significant improvements were noted in physician evaluations after 8 weeks, and after 12 weeks 79% of the patients were found to have an 80% or more improvement. As this was an open-label study, broad conclusions cannot be made concerning the outcome. It is interesting to note, however, that the clinical benefits did not appear until 2 to 3 months after internal consumption of the agent [124]. This implies that the use of internal agents may take time. Indeed oral vitamin E consumption can take weeks before sebum levels are significantly elevated [125]. The addition of mixed carotenoids, presumably inclusive of lycopene, is theoretically sound. Lycopene can reduce lipid peroxidation and it can support SOD and GSH-Px activity [126,127]. Related carotenoids such as lutein and zeaxanthin are also of potential value since they can reduce lipid peroxidation in the skin [128]. It could be speculated that another lipophilic antioxidant, co-enzyme Q10, found in skin surface lipids, would be of therapeutic value [129].

Lactoferrin, a dairy-based glycoprotein with strong antioxidant activity [130,131], has also been the subject of a recent clinical trial in acne. In a 12-week double-blind, placebo-controlled study, the oral consumption of 200 mg of lactoferrin reduced total lesion count by 23% over placebo. The inflammatory lesion count declined by over 38% during the course of the three months. In support of the total lesion reduction, the severity of acne according to the Leeds Acne Grading System declined 20% compared with placebo [132].

The global aspects of diet are also worthy of brief mention. In recent years it has become evident that there may indeed be a connection between dietary components and the risk of acne. For example, regional diets low in processed foods and sugars (with an overall low glycemic load) are associated with decreased acne risk [133]. Intervention studies using similar low glycemic load meals have reported improvements [134]. One of the features of these intervention studies is that they tend to be higher in 'nutrient-dense' foods, and this would include greater intake of plant-based antioxidants from whole grains, vegetables and fruits. Adherence to the so-called South Beach diet, similar to the Mediterranean diet which reduces depression risk by 30%, has been associated with the reduction and discontinuance of acne medications [135].

Here again, we must make note of the connection between oxidative stress, insulin and depression. As mentioned above, induction of oxidative stress has been shown to elevate insulin levels [71], and in turn, insulin itself increases oxidative stress within the skin and steps up demand for SOD [136]. Even in healthy adults, epidemiological studies have made associations between blood chemistry indicative of insulin resistance and an elevated risk of depressive symptoms [137,138]. It has been well documented that a period of insulin resistance occurs during puberty [139], one coinciding with the development of acne, depression and/or anxiety. There is also a generalized increase in SOD activity, 25% higher in a healthy 17-year old vs. a 7-year old [140]. These findings take on greater meaning when placed in the context of recent international studies showing that acne is associated with increased consumption of highly palatable, sweet, fried, calorie-rich foods with low nutrient density [141-143]. Add to this experimental research indicating that regular consumption of such a highly palatable diet increases oxidative stress in the central nervous system and promotes anxiety-like behavior [144].

## Benzoyl Peroxide and Antioxidants

One advantage of oral and/or topical antioxidant interventions may be their ability to augment standard care in acne. An obvious application of antioxidants might be the co-administration with BP, yet there are unanswered questions which must be clarified before individual antioxidants are simply added to BP. Although BP has been used widely for many years in dermatology, and certainly there is evidence that it is helpful in acne, it is not without side effects. It is also not without controversy. In recent years there have been calls to take a closer look at the negative consequences of BP therapy. Experimental studies have shown that BP is cytotoxic and capable of promoting the growth of skin tumors. [145,146] A recent animal study showed that topical BP increased mortality when combined with UV radiation [147]. In one oft-cited study, BP was shown to reduce the experimental production of ROS, although it did so only by killing polymorphonuclear leucocytes that would otherwise induce free radicals [148].

Although BP's role as a tumor promoter has been well established in several animal species, the significance of these findings in humans remains unknown. There are many substances which have potential carcinogenic activity in rodents at high doses and yet do not appear to increase risk or accelerate tumor progression in humans. Thus far, the accumulated evidence, including large-scale population studies, indicates that broad clinical use of BP doses not increase skin cancer risk in humans [149-151]. In our context, BP warrants discussion because its clinical success has been linked with its pro-oxidant activity [152,153]. Indeed, research shows that BP causes major reductions in skin antioxidant levels. Specifically, BP reduces epidermal vitamin E by up to 95% and vitamin C by up to 70% [154].

Given that the sebaceous gland represents the major physiologic route of delivery of one of the most protective agents for human skin - vitamin E - its near elimination by BP should be, at the very least, cause for concern. Sebaceous gland vitamin E secretion has been shown to be highest in areas where it would be advantageous to have additional antioxidant support against environmental assaults (e.g. ratio of facial vs. upper arm epidermal vitamin E levels is 20:1) [155]. These findings would certainly suggest that a simple addition of vitamin E to BP would be rational. However, the co-administration of BP and vitamin E has been found, at least in experimental conditions, to increase total ROS production. In contrast, if vitamin E was used well in advance of BP, then excellent protection against BP toxicity was observed [156]. This study highlights the often-contradictory findings in the area of free radical research, and clearly shows that thorough investigations must be conducted before bench work translates into clinical success. Indeed a combination of vitamin E and the glutathione precursor *N*-acetylcysteine seems to work synergistically in reducing BP toxicity [157]. Obviously there is much work to be done before oral and topical antioxidants can be broadly prescribed in acne. Intervention studies indicating value of topical and oral antioxidant preparations are, at present, far from robust, yet there appears to be more than enough background data to warrant a vigorous pursual of further research.

## Back to the Future

Finally, as a historical note on the old wine, there are older reports showing value of oral vitamins A, C, D, E, and nicotinic acid in acne [158,159]. In today's evidence-based context these publications can only be described as observational or case reports. They are largely from clinicians who followed patients and reported value of certain nutrients over the span of years of clinical experience. Some provided specific details, such as a 1954 report using a combination of 3 grams of ascorbic acid and an 8-ounce glass of citrus juice as reported to be of value in 53 patients with acne. Followed for four months, 43 patients showed improvement, including 15 of which were described as previously treatment resistant [17]. In 1959, a group of dermatologists followed 30 treatment-resistant patients who received 400 mg of ascorbic acid and 400 mg of the flavonoid complex hesperidin (Hesper-C) [18]. The physicians reported that 29 out of the 30 noted definite improvement when Hesper-C was added to other therapies. The researchers concluded the report by stating 'it is hoped that a large clinic will see fit to run a placebo-controlled study to evaluate more critically the effects of hesperidin and ascorbic acid in the treatment of acne.' Over 50 years later, and with more than ample justification, the time may be right to initiate the long-awaited study.

## Competing interests

The authors declare that they have no competing interests.

## Authors' contributions

WPB and ACL contributed equal time and effort in the investigation, research and drafting of this manuscript. All authors read and approved the final manuscript.
